# Polymorphisms of Estrogen Metabolism-Related Genes and Prostate Cancer Risk in Two Populations of African Ancestry

**DOI:** 10.1371/journal.pone.0153609

**Published:** 2016-04-13

**Authors:** Laurent Brureau, Dieudonné Moningo, Elise Emeville, Séverine Ferdinand, Augustin Punga, Simon Lufuma, Pascal Blanchet, Marc Romana, Luc Multigner

**Affiliations:** 1 Inserm, U1085 - IRSET, Pointe-à-Pitre, Guadeloupe, France; 2 Service d’Urologie, CHU de Pointe à Pitre, Pointe à Pitre, Guadeloupe, France; 3 Université des Antilles, Pointe-à-Pitre, Guadeloupe, France; 4 Service d’Urologie, Cliniques Universitaires de Kinshasa, Kinshasa, Democratic Republic of Congo; 5 Université de Rennes 1, Rennes, France; 6 Inserm, U1134, Pointe-à-Pitre, Guadeloupe, France; Ohio State University Medical Center, UNITED STATES

## Abstract

**Background:**

Estrogens are thought to play a critical role in prostate carcinogenesis. It has been suggested that polymorphisms of genes encoding enzymes involved in estrogen metabolism are risk factors for prostate cancer. However, few studies have been performed on populations of African ancestry, which are known to have a high risk of prostate cancer.

**Objective:**

We investigated whether functional polymorphisms of *CYP17*, *CYP19*, *CYP1B1*, *COMT* and *UGT1A1* affected the risk of prostate cancer in two different populations of African ancestry.

**Methods:**

In Guadeloupe (French West Indies), we compared 498 prostate cancer patients and 565 control subjects. In Kinshasa (Democratic Republic of Congo), 162 prostate cancer patients were compared with 144 controls. Gene polymorphisms were determined by the SNaPshot technique or short tandem repeat PCR analysis. Logistic regression was used to estimate adjusted odds ratios (OR) and 95% confidence intervals (CI).

**Results:**

The AA genotype and the A allele of rs4680 (*COMT*) appeared to be inversely associated with the risk of prostate cancer in adjusted models for both Afro-Caribbean and native African men. For the A allele, a significant inverse association was observed among cases with low-grade Gleason scores and localized clinical stage, in both populations.

**Conclusions:**

These preliminary results support the hypothesis that polymorphisms of genes encoding enzymes involved in estrogen metabolism may modulate the risk of prostate cancer in populations of African ancestry.

## Introduction

Prostate cancer is the leading non-cutaneous cancer in men from developed countries, and the second most common such cancer in men worldwide [1]. It disproportionately affects individuals of African ancestry, regardless of their country of residence, and is less common in Caucasian and Asian populations [2]. The reasons for these ethnic differences in incidence are largely unknown, but probably involve complex interplay between hormonal, environmental and genetic factors [2, 3].

There is evidence to suggest that the steroid hormone pathway and genes relating to the metabolism of androgens and estrogens are involved in prostate carcinogenesis [4]. The prostate is an androgen-dependent organ, so many studies initially focused on the influence of androgens. However, attention has increasingly focused on the role of estrogen in prostate cancer, in during recent years. It is now suspected that exposure to endogenous or environmental estrogens may contribute to prostate gland carcinogenesis and that the decrease in the androgen/estrogen ratio with aging may play a role in this process [5]. Estrogens, though their receptors or catechol metabolites, have been identified as potential carcinogens [6, 7]. Based on these observations, functional polymorphisms of genes related to estrogens metabolism pathways have been proposed as candidates for association studies on prostate cancer risk [8]. Various studies have investigated the associations between polymorphisms of genes encoding enzymes involved in estrogen metabolism and the risk of prostate cancer [9]. However, most such studies focused on Caucasian or Asian populations; very few have investigated populations of African ancestry which are recognized to be at higher risk of the disease.

In this study we focused on several common functional polymorphisms of key genes encoding enzymes involved in estrogen metabolism, biosynthesis and catabolism: *CYP17*, *CYP19*, *CYP1B1*, *COMT*, and *UGT1A1*.

The *CYP17* gene encodes cytochrome P450c17, which catalyzes the conversion of 17-hydroxypregnenolone and 17-hydroxyprogesterone to dehydroepiandrosterone and androstenedione, respectively. A polymorphism (rs743572) in the 5’-untranslated promoter region causes the replacement of a thymidine (T) with a cytosine (C) at nucleotide 34. The C variant allele (also known as A2) is associated with higher levels of enzymatic activity, and, thus, with higher circulating levels of estradiol than the wild-type T allele (also known as A1) [10]. The *CYP19* gene encodes aromatase, an enzyme that converts androstenedione and testosterone to estrone and estradiol, respectively. A microsatellite polymorphism (rs60271534) consisting of tandem (TTTA)n repeats in intron 4 has been described. Associations between the number of repeats and the level of circulating estrogens have been reported [11]. The *CYP1B1* gene encodes cytochrome P4501B1, an enzyme principally catalyzing the addition of a 4-hydroxyl group to estrone and estradiol. A cytosine (C) to guanine (G) substitution (rs1056836) in exon 3 results in the replacement of a valine residue with a leucine residue at codon 434. The valine variant protein has been shown to be associated with higher levels of catalytic activity than the wild-type leucine variant [12]. The *COMT* gene encodes catechol-O-methyl-transferase, which converts catechol estrogens into inactive metabolites. A guanine (G) to alanine (A) substitution (rs4680) in exon 7 result in the replacement of a valine residue with a methionine residue at codon 158, this change being associated with a three- to four-fold decrease in activity related to a decreased thermolability of the protein [13]. Furthermore, it has been shown that the A allele (Met158) was more highly expressed in human lymphoblast lines and brain than the G allele [14]. UGT1A1 catalyzes the glucuronidation of estrogen metabolites, facilitating their excretion. A functional microsatellite polymorphism (rs8175347) consisting of dinucleotide (TA)_n_ repeats located in the TATA box of the gene has been identified. Larger numbers of repeats are associated with lower levels of transcription and, thus, lower levels of glucuronidation activity [15, 16].

We investigated the associations between the five gene polymorphisms described above and the risk of prostate cancer in two different populations of African origin: an Afro-Caribbean population from Guadeloupe (French West Indies) and a native African population from Kinshasa (Democratic Republic of Congo).

## Materials and Methods

### Populations Study

This study was conducted at two locations, one in the Caribbean (Guadeloupe) and the other in Central Africa (Kinshasa, Democratic Republic of Congo). Guadeloupe is a Caribbean archipelago of five islands covering 1628 square km, with around 404,000 inhabitants. It is a French overseas *département* and is thus part of the European Union, with a gross domestic product per capita of about US$ 21000 in 2012 [17]. The world age-standardized incidence of prostate cancer in Guadeloupe was estimated at 186 per 100,000 men in 2006 [18], an incidence similar to those reported for African-American men in the US and Afro-Caribbean or African men in the United Kingdom [19, 20]. Most of the inhabitants (90%) of Guadeloupe are the descendants of slaves from West and Central Africa. Kinshasa is the capital of the Democratic Republic of the Congo. It has a population of over 12 million. The gross domestic product per capita was estimated at US$ 391 in 2012 [21]. In the absence of a cancer registry for Kinshasa, the International Agency for Research on Cancer estimated the incidence of prostate cancer in the Democratic Republic of the Congo at about 31 cases per 100,000 men [22].

In Guadeloupe, we conducted a population-based case-control study including 498 consecutive incident cases of histologically confirmed prostate cancer, and 565 controls without prostate cancer from 2005 to 2007. The selection of cases and controls has been described elsewhere [23]. Briefly, cases were recruited from public and private urology clinics in a recruitment area covering the entire territory of the Guadeloupe Archipelago. Controls were recruited from men participating in a free systematic health screening program open to the general population: each year, a random population sample, selected in accordance with the sex and age distribution of the general population, was invited to participate. Consecutive men aged 45 years and over attending the screening program were invited to participate, with selection according to the approximate age distribution of prostate cancer incidence in Guadeloupe. The inclusion criteria for both cases and controls were current residence in Guadeloupe, both parents born on any Caribbean island with a population of predominantly African descent. Additional inclusion criteria for controls were normal findings upon digital rectal examination and total plasma PSA concentration no higher than the 75th percentile for the corresponding age group of African-American men with no clinical evidence of prostate cancer [24].

In Kinshasa, we conducted a hospital-based case-control study at the University Clinic of Kinshasa. We included 162 consecutive incident cases of histologically confirmed prostate cancer and 144 controls without prostate cancer from 2011 to 2013. Control subjects were recruited consecutively from men attending for prostate cancer screening or benign prostatic hyperplasia. For subjects taking part in prostate cancer screening, we retained the criteria described above (digital examination and PSA concentrations) for the Guadeloupean control population. All subjects with benign prostatic hyperplasia underwent a histological evaluation of prostate tissue after transurethral resection of the prostate; an absence of malignancy was confirmed in all cases. The inclusion criteria for both patients and controls were birth in a sub-Saharan African country, with both parents also born in sub-Saharan African countries.

In both populations, cases and controls were excluded if they presented past or current cancer (except for prostate cancer, for cases), past or current treatment with 5-alpha reductase inhibitors or any drug known to influence the hypothalamic-pituitary-gonadal-adrenal axis. All subjects were interviewed in person, to obtain information about their age, their own and their parents’ places of birth, education, weight and height, for the calculation of body mass index (BMI, kg/m^2^), smoking, alcohol consumption, family history of prostate cancer, and diabetes. Studies were approved by the Guadeloupean ethics committee for studies involving human subjects and by the ethics committee of the University Clinic of Kinshasa. Each participant provided written informed consent.

### Genotyping analysis

In Guadeloupe, participants provided blood in conventional EDTA tubes. In Kinshasa, participants provided saliva samples using the Oragene^®^ OG-300 kit (DNA Genotek), and then transferred to Guadeloupe where all following analysis were done. Genomic DNA was extracted from peripheral blood leukocytes by standard procedures or from saliva according the manufacturer procedure. DNA was quantified with a NanoVue Plus^™^ (GE Healthcare Bio-Sciences, Uppsala, Sweden). Single-nucleotide polymorphisms of the *CYP17*, *CYP1B1* and *COMT* genes were screened by multiplex PCR amplifications followed by SNaPshot single-nucleotide primer extension. Briefly, PCR was performed as follows: initial denaturation for 5 min at 95°C, followed by 35 cycles of 95°C for 1 min, 62°C for 45 seconds and 72°C for 45 seconds. PCR products were subjected to electrophoresis in a 2% agarose gel, and purified with exonuclease I and shrimp alkaline phosphatase (USB Corporation, High Wycombe, UK) treatment to remove unincorporated deoxynucleotides and primers from the amplified DNA. For primer extension with target complementary fluorescent dideoxyribonucleotides (ddNTPs), SNaPshot reactions were performed using the SNaPshot^®^ Multiplex Kit (Applied Biosystems, Warrington, UK). The labeled extension products were analyzed by capillary electrophoresis. The number of *UGT1A1* and *CYP19* tandem repeat sequences was determined by duplex PCR on a Perkin-Elmer 2700 thermocycler (Perkin-Elmer Corporation, Norwalk, USA). PCR amplification was carried out as follows: 95°C for 5 minutes, followed by 35 cycles of 95°C for 30 seconds, 55°C for 40 seconds and 72°C for 45 seconds. PCR products were then subjected to electrophoresis in a 2% agarose gel and analyzed with an ABI 3130 DNA sequencer (Applied Biosystems, Foster City, USA). Genotyping was carried out blind to the case-control status of the subject. For quality control, a subsample of 10% of the subjects studied were genotyped twice.

### Statistical analysis

We checked the consistency of all the genotypes studied in our analysis of bi-allelic polymorphism for consistency with Hardy-Weinberg equilibrium in the control population. Standard chi-squared tests were used to compare genotype and allele frequencies between the case and control groups. Odds ratios (ORs) and 95% confidence intervals (CIs) for the associations between gene polymorphisms and prostate cancer were estimated by unconditional logistic regression. We used univariate logistic regression analysis to evaluate potential confounding factors (age, parents’ place of birth, education, body mass index, waist-to-hip-ratio, smoking habits, alcohol consumption, type 2 diabetes, family history of prostate cancer, PSA screening history). Confounders were selected on the basis of change-in-estimate criterion if their inclusion in the model modified the estimated unadjusted OR by >10% [25]. Age and education (as surrogate of socioeconomic status) were systematically included in the adjusted model. Log linearity was not achieved for age, so this variable was categorized as quartiles, according to the age distribution of controls. Missing data for covariates were handled using a dummy variable.

For allele analyses, *CYP17*, *CYP1B1*, and *COMT* were coded as counts of risk alleles (wt = 0; var = 1) and *CYP19* and *UGT1A1* were classified according to the number of repeats (≤7 = 0; >7 = 1) and (≤6 = 0; >6 = 1) as previously reported, respectively [16, 26]. For genotype analyses, *CYP17*, *CYP1B1*, and *COMT* were coded (wt/wt = 0; wt/var = 1 and var/var = 2) and *CYP19* and *UGT1A1* s were classified according to the number of repeats (≤7/≤7 = 0; ≤7/>7 = 1; >7/>7 = 2) and (≤6/≤6 = 0; ≤6/>6 = 1; >6/>6 = 2), respectively.

Tests for trend in risk were performed by entering the categorical variable into the model as ordinal variables. Polytomous logistic regressions were used to estimate risk simultaneously in controls and non-ordered subgroups of cases, as a function of grade (low grade: Gleason score <7 or 3+4; high grade: Gleason score 4+3 or >7) or clinical stage at diagnosis (tumor, nodes, metastases; localized stage: T1c or T2 and N0 and M0; advanced stage: T3 or T4, or N+ or M+). These analyses were restricted to alleles, to decrease the sampling fluctuation due to the presence of small numbers of cases in strata. Statistical analyses were carried out with SAS software version 9.3 (SAS Institute, Cary, NC). All tests were two-tailed, and *P* values less than 0.05 were considered significant.

## Results

The general characteristics of the study participants are summarized in Table 1. The most notable differences between the two populations studied were the higher level of education (in both controls and cases), and the higher Gleason score and clinical stage (cases) in Congolese than in Guadeloupean subjects. We also noted that there was a difference in the percentage of individuals with a family history of prostate cancer between cases and controls for the Guadeloupean, but not for the Congolese population.

**Table 1:** Baseline characteristics of the study populations.

Characteristics	Afro-Caribbean			Native African		
	Cases (*n* = 498)	Controls (*n* = 565)	*P* ^a^	Cases (*n* = 162)	Controls (*n* = 144)	*P* ^a^
**Age** (mean, range)	66.4 (45.8–94.5)	60.4 (45.1–88.8)	<0.001	68.9 (43.2–87.6)	63.7 (42.1–85.5)	<0.001
**PSA** (mean, range)	30.4 (0.84—>1000)	1.1 (0.04–4.9)	-	77.5 (3.0–936)	2.4 (0.2–6.5)	-
**Parents’ place of birth** (*n*, %)						
French West Indies	479 (96.2)	518 (91.7)		-	-	
Other Caribbean islands ^b^	19 (3.8)	47 (8.3)	0.002	-	-	-
Congo Kinshasa	-	-	-	155 (95.7)	133 (92.4)	
Other African countries ^c^	-	-	-	7 (4.3)	11 (7.6)	0.22
**Education** (*n*, %)						
Primary	308 (62.4)	301 (56.0)		42 (25.9)	14 (9.7)	
Secondary	124 (25.1)	175 (32.6)		44 (27.2)	25 (17.4)	
High school or above	62 (12.5)	61 (11.4)	0.03	76 (46.9)	105 (72.9)	<0.001
**Body mass index** (kg/m^2^) (*n*, %)						
< 25	199 (42.7)	258 (45.7)		102 (63.0)	70 (48.6)	
25—< 30	206 (44.2)	237 (42.0)		47 (29.0)	54 (37.5)	
≥ 30	61 (13.1)	69 (12.2)	0.62	13 (8.0)	20 (13.9)	0.03
**Waist-to-hip-ratio** (*n*, %)						
≤ 0.95	182 (55.0)	392 (69.7)		117 (72.2)	99 (68.8)	
> 0.95	149 (45.0)	170 (30.2)	<0.001	45 (27.8)	45 (31.3)	0.51
**Smoking** (*n*, %)						
Never	309 (62.7)	345 (61.4)		103 (63.6)	90 (62.9)	
Former or current smoker	184 (37.3)	217 (38.6)	0.67	59 (36.4)	53 (37.1)	0.91
**Alcohol consumption** (*n*, %)						
Never	68 (13.9)	80 (14.4)		26 (16.0)	27 (18.8)	
Former or current drinker	422(86.1)	474 (85.6)	0.79	136 (84.0)	117 (81.3)	0.53
**Type 2 diabetes** (*n*, %)						
No	400 (82.6)	491 (88.9)		148 (91.4)	132 (91.7)	
Yes	84 (17.4)	61 (11.1)	0.003	14 (8.6)	12 (8.3)	0.92
**Family history of prostate cancer** (*n*, %)						
No	269 (54.8)	429 (78.4)		116 (71.6)	111 (77.1)	
Yes	113 (23.0)	61 (11.2)		22 (13.6)	20 (13.9)	
Don’t know	109 (22.2)	57 (10.4)	<0.001	24 (14.8)	13 (24.0)	0.30
**PSA screening history** (*n*, %)						
No	242 (48.9)	489 (86.5)		-	-	
Yes	253 (51.1)	76 (13.5)	<0.001	-	-	-
**Gleason score**						
< 7 or 3 + 4	395 (79.3)	-		63 (38.9)	-	
> 7 or 4 + 3	90 (18.6)	-		88 (54.3)	-	
Not provided	13 (2.1)	-		11 (6.8)	-	
**TNM**						
T1c or T2 and N0 and M0	417 (83.7)	-		94 (58.0)	-	
T3 or T4, or N+ or M+	63 (12.7)	-		68 (42.0)	-	
Not provided	18 (3.6)	-		-	-	

^**a**^ *P* values were calculated in two-tailed Chi^2^ tests for comparisons of percentages and in two-tailed Student’s *t* tests or Mann-Whitney tests for comparisons of means,

^**b**^ Dominica, Haiti,

^**c**^ Angola, Cameroon, Ivory Coast, Mali, Niger, Tanzania

The distributions of genotypes and alleles for the *CYP17*, *CYP19*, *CYP1B1*, *COMT* and *UGT1A1* polymorphisms in controls and prostate cancer patients are shown in Table 2. Each of the three single-nucleotide polymorphisms was in Hardy-Weinberg equilibrium in controls from both populations (data not shown). Detailed genotype and allele distributions for *CYP19* and *UGT1A1* in cases and controls are presented in S1 and S2 Tables respectively.

**Table 2:** Genotypes and allele frequencies in cases and controls.

Gene	Afro-Caribbean		Native African	
	Cases Frequency (*n*)	Controls Frequency (*n*)	Cases Frequency (*n*)	Controls Frequency (*n*)
***CYP17* (rs743572)**				
**Genotypes**				
T/T	0.41 (178)	0.39 (208)	0.37 (55)	0.36 (50)
T/C	0.46 (199)	0.50 (265)	0.47 (71)	0.49 (68)
C/C	0.13 (57)	0.11 (59)	0.16 (24)	0.15 (21)
**Alleles**				
T	0.64 (555)	0.65 (681)	0.61 (182)	0.60 (168)
C	0.36 (309)	0.35 (375)	0.39 (118)	0.40 (110)
***CYP19* (rs60271534)**				
**Genotypes**				
≤7/≤7	0.64 (272)	0.63 (329)	0.66 (97)	0.65 (89)
≤7/>7	0.32 (137)	0.32 (168)	0.31 (46)	0.34 (47)
>7/>7	0.04 (17)	0.05 (25)	0.03 (4)	0.02 (2)
**Alleles**				
≤7	0.80 (681)	0.79 (826)	0.82 (240)	0.82 (225)
>7	0.20 (171)	0.21 (218)	0.18 (54)	0.18 (51)
**CYP1B1 (rs1056836)**				
**Genotypes**				
C/C	0.05 (24)	0.08 (42)	0.02 (3)	0.02 (3)
C/G	0.40 (180)	0.37 (205)	0.28 (42)	0.31 (43)
G/G	0.55 (252)	0.55 (301)	0.70 (105)	0.67 (93)
**Alleles**				
C	0.25 (228)	0.26 (289)	0.16 (49)	0.18 (49)
G	0.75 (684)	0.77 (807)	0.84 (251)	0.82 (229)
***COMT* (rs4680)**				
**Genotypes**				
G/G	0.45 (203)	0.42 (229)	0.61 (91)	0.50 (69)
G/A	0.48 (220)	0.46 (254)	0.36 (54)	0.42 (58)
A/A	0.07 (33)	0.12 (65)	0.03 (5)	0.08 (12)
**Alleles**				
G	0.69 (626)	0.65 (712)	0.79 (236)	0.71 (196)
A	0.31 (286)	0.35 (384)	0.21 (64)	0.29 (82)
***UGT1A1* (rs8175347)**				
**Genotypes**				
≤6/≤6	0.32 (140)	0.31 (161)	0.31 (45)	0.27 (38)
≤6/>6	0.45 (197)	0.50 (266)	0.44 (65)	0.46 (63)
>6/>6	0.23 (99)	0.19 (99)	0.25 (37)	0.27 (37)
**Alleles**				
≤6	0.56 (588)	0.55 (477)	0.53 (155)	0.50 (139)
>6	0.44 (464)	0.45 (395)	0.47 (139	0.50 (137)

*n*: total number of individuals and total number of chromosomes for genotype frequency and allele frequency, respectively.

A comparison of the frequency distribution of genotypes and alleles in our control populations is presented in S3 Table. Some differences between Congo and Guadeloupe were observed for *CYP1B1* and *UGT1A1*. However, in both populations, all gene frequencies were within the range previously reported for populations of African ancestry [20].

No association was found between *CYP17*, *CYP19*, *CYP1B1* and *UGT1A1* polymorphisms and prostate cancer risk in crude and adjusted models, for either the Afro-Caribbean or native African population (Table 3). By contrast, the homozygous AA genotype of rs4680 (*COMT*) appeared to be inversely associated with prostate cancer risk in adjusted models, for both Afro-Caribbean (OR: 0.53; 95% CI: 0.32–0.88; P_trend_: 0.04) and native African (OR: 0.26, 95% CI: 0.08–0.83; P_trend_: 0.003) populations. Similar significant inverse associations were observed for the A allele (OR: 0.81, 95% CI: 0.67–0.99 and OR: 0.54, 95% CI: 0.35–0.82, for the Afro-Caribbean and native African populations, respectively).

**Table 3:** Association between polymorphisms of estrogen metabolism-related genes and the risk of prostate cancer.

	Afro-Caribbean			Native African		
	Cases/Controls *n*	Crude OR (95% CI)	Adjusted OR ^a^ (95% CI)	Cases/Controls *n*	Crude OR (95% CI)	Adjusted OR ^b^ (95% CI)
***CYP17* (rs743572)**						
**Genotypes**						
T/T	178/208	1.0	1.0	55/50	1.0	1.0
T/C	199 /265	0.88 (0.67–1.15)	0.80 (0.59–1.07)	71/68	0.95 (0.57–1.58)	0.94 (0.54–1.66)
C/C	57/59	1.13 (0.74–1.71)	1.00 (0.64–1.57)	24/21	1.04 (0.52–2.09)	1.25 (0.58–2.70)
*p-*trend		0.98	0.53		0.98	0.72
**Alleles**						
T	555/681	1.0	1.0	182/168	1.0	1.0
C	309/375	1.01 (0.84–1.22)	0.95 (0.77–1.16)	118/110	0.99 (0.71–1.38)	1.06 (0.73–1.54)
***CYP19* (rs60271534)**						
**Genotypes**						
≤7/≤7	272/329	1.0	1.0	97/89	1.0	1.0
≤7/>7	137/168	0.99 (0.75–1.30)	0.86 (0.61–1.20)	46/47	0.90 (0.55–1.48)	0.85 (0.48–1.49)
>7/>7	17/25	0.82 (0.43–1.55)	0.67 (0.28–1.60)	4/2	1.84 (0.33–10.3)	0.80 (0.13–5.02)
*p-*trend		0.67	0.24		0.97	0.49
**Alleles**						
≤7	681/826	1.0	1.0	240/225	1.0	1.0
>7	171/218	0.95 (0.76–1.19)	0.85 (0.64–1.12)	54/51	0.99 (0.65–1.52)	0.89 (0.55–1.44)
***CYP1B1* (rs1056836)**						
**Genotypes**						
C/C	24/42	1.0	1.0	105/93	1.0	1.0
C/G	180/205	1.54 (0.89–2.64)	1.09 (0.54–2.23)	42/43	0.87 (0.52–1.44)	0.91 (0.52–1.60)
G/G	252/301	1.46 (0.86–2.48)	1.10 (0.55–2.20)	3/3	0.89 (0.17–4.50)	0.46 (0.08–2.72)
*p*-trend		0.48	0.84		0.59	0.37
**Alleles**						
C	228/289	1.0	1.0	251/229	1.0	1.0
G	684/807	1.07 (0.88–1.31)	1.01 (0.77–1.31)	49/49	0.91 (0.59–1.41)	0.86 (0.53–1.39)
***COMT* (rs4680)**						
**Genotypes**						
G/G	203/229	1.0	1.0	91/69	1.0	1.0
G/A	220/254	0.98 (0.75–1.26)	0.95 (0.72–1.26)	54/58	0.71 (0.43–1.15)	0.53 (0.30–0.91)
A/A	33/65	0.57 (0.36–0.91)	0.53 (0.32–0.88)	5/12	0.32 (0.11–0.94)	0.26 (0.08–0.83)
*p*-trend		0.07	0.04		0.02	0.003
**Alleles**						
G	626/712	1.0	1.0	236/196	1.0	1.0
A	286/384	0.85 (0.70–1.02)	0.81 (0.67–0.99)	64/82	0.65 (0.44–0.95)	0.54 (0.35–0.82)
***UGT1A1 (*rs8175347)**						
**Genotypes**						
≤6/≤6	140/161	1.0	1.0	45/38	1.0	1.0
≤6/>6	197/266	0.85 (0.63–1.14)	1.02 (0.71–1.47)	65/63	0.87 (0.50–1.52)	1.22 (0.66–2.26)
>6/>6	99/99	1.15 (0.80–1.65)	1.52 (0.98–2.35)	37/37	0.84 (0.45–1.58)	0.91 (0.46–1.82)
*p*-trend		0.56	0.05		0.59	0.84
**Alleles**						
≤ 6	477/588	1.0	1.0	155/139	1.0	1.0
> 6	395/464	1.05 (0.88–1.26)	1.22 (0.98–1.53)	139/137	0.91 (0.66–1.26)	0.96 (0.66–1.39)

^**a**^ Adjusted for age and education for *CYP17* and *COMT*; for age, waist-to-hip-ratio and education for *CYP19*; for age, waist-to-hip-ration, type 2 diabetes, BMI, education and PSA screening history for *CYP1B1*; for age, education and waist-to-hip-ratio for *UGT1A1*.

^**b**^ Adjusted for age for *CYP17* and *COMT*; for age, BMI and alcohol consumption for *CYP19*; for age and education for *CYP1B1* and for *UGT1A1*.

We investigated the dependence of the gene polymorphism-prostate cancer association on clinical characteristics in the Afro-Caribbean (Table 4) and native African populations (Table 5). No significant associations were found for *CYP17*, *CYP19* or *CYP1B1*, for Gleason score or clinical stage. The A allele of rs4680 (*COMT)* was inversely associated with low-grade Gleason scores (OR: 0.80, 95% CI: 0.64–0.98) and localized clinical stage (OR: 0.82, 95% CI: 0.67–1.01), in Afro-Caribbean men. Similar inverses associations were observed in the native African population (OR: 0.35, 95% CI: 0.19–0.65 and OR: 0.45, 95% CI: 0.25–0.75 for low-grade Gleason score and localized clinical stage, respectively). Finally, the presence of long (TA)n repeats (*n*>6) in *rs8175347 (UGT1A1*) was significantly associated with a high Gleason score (OR: 1.48, 95% CI: 1.03–2.11) and advanced clinical stage (OR: 1.59, 95% CI: 1.04–2.43) in the Afro-Caribbean population (Table 4), but not in the native African population (Table 5).

**Table 4:** Association between polymorphisms of estrogen metabolism-related genes related to estrogen metabolism and the risk of prostate cancer, by Gleason score and clinical stage, in the Afro-Caribbean population.

Genes (allele)		Gleason Score				Clinical Stage			
		Low Grade		High Grade		Localized		Advanced	
	Controls *n* (%)	Cases *n* (%)	Adjusted OR ^a^ (95% CI)	Cases *n* (%)	Adjusted OR ^a^ (95% CI)	Case *n* (%)	Adjusted OR ^a^ (95% CI)	Cases *n* (%)	Adjusted OR ^a^ (95% CI)
***CYP17*(rs743572)**									
T	681 (64.5)	434 (63.4)	1.0	106 (65.8)	1.0	476 (65.4)	1.0	68 (59.6)	1.0
C	375 (35.5)	251 (36.4)	0.99 (0.80–1.22)	55 (34.2)	0.86 (0.60–1.24)	252 (34.6)	0.92 (0.75–1.13)	46 (40.4)	1.06 (0.69–1.63)
***CYP19* (rs60271534)**									
≤7	826 (79.1)	540 (80.8)	1.0	129 (77.7)	1.0	562 (79.6)	1.0	94 (81.0)	1.0
>7	218 (20.9)	128 (19.2)	0.80 (0.60–1.08)	37 (22.3)	0.99 (0.64–1.53)	144 (20.4)	0.84 (0.63–1.12)	22 (19.0)	0.83 (0.48–1.42)
***CYP1B1* (rs1056836)**									
C (Leu)	289(26.4)	177 (24.3)	1.0	46 (28.1)	1.0	188 (24.4)	1.0	34(29.3)	1.0
G (Val)	807(73.6)	551 (75.7)	1.04 (0.79–1.37)	118 (71.9)	0.89 (0.59–1.35)	585 (75.6)	1.04 (0.79–1.37)	82 (70.7)	0.80 (0.49–1.30)
***COMT* (rs4680)**									
G (Val)	712 (65.0)	506 (69.5)	1.0	107 (65.2)	1.0	527 (68.4)	1.0	83 (71.5)	1.0
A (Met)	384 (35.0)	222 (60.5)	0.80 (0.64–0.98)	57 (34.8)	0.95 (0.66–1.35)	243 (31.6)	0.82 (0.67–1.01)	33 (28.5)	0.68 (0.43–1.07)
***UGT1A1* (rs8175347)**									
≤ 6	588 (55.9)	377 (55.3)	1.0	86 (50.6)	1.0	404 (55.8)	1.0	56 (46.7)	1.0
> 6	464 (44.1)	305 (44.7)	1.20 (0.96–1.51)	84 (49.4)	1.48 (1.03–2.11)	320 (44.2)	1.19 (0.95–1.49)	64 (53.3)	1.59 (1.04–2.43)

^**a**^ Adjusted for age and education for *CYP17* and *COMT*; for age, waist-to-hip-ratio and education for *CYP19*; for age, waist-to-hip-ration, type 2 diabetes, BMI, education and PSA screening history for *CYP1B1*; for age, education and waist-to-hip-ratio for *UGT1A1*.

**Table 5:** Association between polymorphisms of estrogen metabolism-related genes and the risk of prostate cancer, by Gleason score and clinical stage, for the native African population.

		Gleason Score				Clinical Stage			
		Low Grade		High Grade		Localized		Advanced	
	Controls *n* (%)	Cases *n* (%)	Adjusted OR ^a^ (95% CI)	Cases *n* (%)	Adjusted OR ^a^ (95% CI)	Cases *n* (%)	Adjusted OR ^a^ (95% CI)	Cases *n* (%)	Adjusted OR ^a^ (95% CI)
***CYP17* (rs743572)**									
T (A1)	182 (60.7)	65 (60.2)	1.0	103 (60.6)	1.0	102 (60.0)	1.0	81 (61.4)	1.0
C (A2)	118 (39.3)	43 (39.8)	1.08 (0.67–1.75)	67 (39.4)	1.08 (0.70–1.66)	68 (40.0)	1.10 (0.72–1.68)	51 (38.6)	1.01 (0.64–1.60)
***CYP19* (rs60271534)**									
≤7	240 (81.6)	88 (86.3)	1.0	136 (80.0)	1.0	136 (81.0)	1.0	106 (82.8)	1.0
>7	54 (18.4)	14 (13.7)	0.68 (0.35–1.32)	34 (20.0)	1.03 (0.60–1.7-)	32 (19.0)	0.94 (0.55–1.60)	22 (17.2)	0.82 (0.45–1.49)
***CYP1B1* (rs1056836)**									
G (Val)	251 (83.7)	93 (86.1)	1.0	141 (82.9)	1.0	147 (86.5)	1.0	105 (79.5)	1.0
A (Leu)	49 (16.3)	15 (13.9)	0.78 (0.41–1.50)	29 (17.1)	0.88 (0.51–1.54)	23 (13.5)	0.71 (0.40–1.26)	27 (20.5)	1.10 (0.63–1.94)
***COMT* (rs4680)**									
G (Val)	196 (70.5)	92 (85.2)	1.0	130 (76.5)	1.0	137 (81.5)	1.0	99 (75.0)	1.0
A (Met)	82 (29.5)	16 (14.8)	0.35 (0.19–0.65)	140 (23.5)	0.63 (0.38–1.02)	131 (18.5)	0.45 (0.27–0.75)	33 (25.0)	0.68 (0.41–1.14)
***UGT1A1* (rs8175347)**									
≤ 6	155 (52.7)	57 (55.9)	1.0	57 (55.9)	1.0	66 (51.6)	1.0	90 (53.6)	1.0
> 6	139 (47.3)	45 (44.1)	0.86 (0.54–1.39)	45 (44.1)	1.06 (0.69–1.62)	62 (48.4)	0.94 (0.62–1.42)	78 (46.4)	1.00 (0.64–1.57)

^**a**^ Adjusted for age for *CYP17* and *COMT*; for age, BMI and alcohol consumption for *CYP19*; for age and education for *CYP1B1* and for *UGT1A1*.

## Discussion

We report here the results of a study of the association of polymorphisms of five estrogen-related genes to prostate cancer risk in two different populations of African ancestry, an Afro-Caribbean population from the French West Indies and a native African population from the Democratic Republic of Congo.

We assumed a common sub-Saharan African ancestry for the two populations studied. Ethnic identification is always difficult and misclassifications due to mixed ancestry can never be excluded. In Guadeloupe, at least 90% of the inhabitants are descended from slaves originating from West and Central Africa. The remaining 10% of the population are descended from Indian immigrants arriving during the 19^th^ century or from more recent immigrants arriving from the Middle East and Europe in the 20^th^ century. We included only subjects whose parents were born in the French West Indies or in two other Caribbean islands with a population of predominantly African ancestry (Haiti, Dominica). In Kinshasa, we included only subjects whose parents were born in a sub-Saharan country. Moreover, although some differences in genotype distribution were found between our two populations (S3 Table), the genotype frequencies in the two populations were within the range previously reported for populations of African ancestry [27] and thus gave us some confidence in the African ancestry of the two populations under study.

The two populations studied had similar ancestral geographic origins, but they differed markedly in terms of socioeconomic and environmental conditions. The French West Indies consists of well-developed territories belonging to the European Union in the Caribbean, with schooling for the entire population and free access to healthcare. By contrast, the Democratic Republic of Congo is a low-income Central African country in which access to education and healthcare is limited. Despite these differences, the population from Kinshasa studied here included men with a high level of education, reflecting the restriction of access to healthcare to people with a certain level of wealth. We also noted that a very high percentage of the prostate cancer cases were diagnosed at an advanced stage of disease. We cannot rule out the possibility that there is an inherently high prevalence of an aggressive form of the disease in this population, but we believe that this finding is more likely to be due to the low level of early prostate cancer detection by PSA screening. Our results should be seen in light of these considerations.

Seven small studies (including 8 to 132 prostate cancer cases) have explored the relationship between the rs743572 (*CYP17*) polymorphism and the risk of prostate cancer in populations of African ancestry. Six of these studies were carried out in the US [28–33], the remaining study being performed in Brazil [34]. Three meta-analyses based on these studies reported that the C allele or the CC genotype was marginally, but not significantly associated with prostate cancer risk [35–37]. Our results indicate that the C allele and the CC genotype are not associated with a significant risk of prostate cancer in either Afro-Caribbean or native African men. Moreover, no associations were found with Gleason score or clinical stage in cases. In other ethnic groups (Caucasian and Asian), no significant association was found between the rs743572 (*CYP17*) polymorphism and prostate cancer [35].

To our knowledge, this is the first study to have explored the relationship between the rs60271534 (*CYP19)* polymorphism and prostate cancer in populations of African ancestry. We found no significant association between prostate cancer and the number of (TTTA)_n_ repeats, regardless of the type of modeling used (allele or genotype analysis), in either Afro-Caribbean or native African populations. We also found no association between the number of (TTTA)_n_ repeats and Gleason score or clinical stage, in the Afro-Caribbean and native African populations studied. Using a sibling-based design, Li *et al*. [38] found no effect of (TTTA)_n_ repeat number on prostate cancer risk in Caucasian populations, whereas Cussenot *et al*. [8] reported an association between larger numbers of (TTTA)_n_ repeats (n>8) and prostate cancer in Caucasians, particularly among patients with early disease onset. In Japanese populations, genotypes with fewer than nine TTTA repeats (*n*<9) have been shown to be associated with a familial risk of prostate cancer [39], whereas alleles with more than seven repeats are associated with poorer cancer-specific survival in patients with bone metastasis at diagnosis [26].

The association of the rs1056836 (*CYP1B1)* polymorphism with the risk of prostate cancer has been intensively investigated in Caucasian and Japanese populations. A recent meta-analysis including 3,221 cases and 3,447 controls from 10 case-control studies provided evidence for a lack of association of the GG variant genotype with prostate cancer risk overall, except in Asians, in studies stratified by ethnicity [40]. However, in a large study of French Caucasians, the G variant allele was found to be significantly associated with a risk of prostate cancer, particularly in cases with aggressive forms [8]. In the only study performed to date in African-Americans, no difference in allele frequencies was reported between prostate cancer cases and controls [32]. Our findings confirm the lack of association between this polymorphism and the risk of prostate cancer in Afro-Caribbean and native African populations. Furthermore, no differences as a function of Gleason score or clinical stage were observed in either population.

The association of the *COMT* (rs4680) polymorphism with the risk of prostate cancer has been investigated only in Caucasian and Asian populations, and three meta-analyses have reported no overall association [41–43]. However, one of these studies reported a significant inverse association in Asian carriers of the A allele [43]. We show here that the AA genotype or A allele was associated with a significantly lower risk of prostate cancer in Afro-Caribbean and native African populations. This inverse association appeared to be more pronounced in cases with a low Gleason score or a localized tumor than in those with a high Gleason score or advanced disease. At first glance, this protective effect is counterintuitive, because the A allele of *COMT* is believed to be associated with a lower efficiency (by a factor of three to four) of carcinogenic catechol estrogen (2- and 4-hydroxyestrogens) conversion into inactive metabolites. The similar inverse associations observed in our two study populations make it less likely that this observation was due to chance, although this remains possible. Green tea-drinking women with the homozygous AA genotype of *COMT* have been shown to have a lower risk of breast cancer [44]. *COMT* encodes an enzyme known to be involved in the rapid O-methylation of factors protective against tumorigenesis, such as the cathechin polyphenols present in green tea [45]. Carriers of the AA variant excrete smaller amounts of polyphenols in their urine [45], suggesting that they retain larger amounts of polyphenols and derive greater health benefits from green tea intake [46]. Neither of the populations studied here has a particularly high level of tea consumption, but an as yet unidentified protective dietary compound metabolized by the COMT pathway may be present. We cannot exclude the possibility that our findings result from the complexity of estrogen signaling due to the functional heterogeneity of estrogen receptors and/or multiple gene-gene interactions.

Two studies on Caucasian populations have investigated the association between *UGT1A1* promoter region polymorphism (TA)_n_ and prostate cancer risk [47, 48]. Overall, no significant association, but one study reported that heterozygosity (≤6/>6 repeats) but not homozygosity (>6/>6 repeats) for the variant number of repeats was significantly more strongly associated with the risk of low-grade prostate cancer than the ≤6/≤6 genotype [48]. Here we found that, in Afro-Caribbean men, alleles with large numbers of repeats (>6) were associated with a higher risk of prostate cancer (marginal significance) than short (≤6 repeats) alleles. Long alleles were also significantly associated with high-grade and advanced-stage prostate cancer. Long repeats might be associated with low levels of catalytic activity and lower levels of tumorigenic estrogen metabolite inactivation. However, these findings were not replicated in the native African population. Thus, the association between UGT1A1 genotype and prostate cancer in Afro-Caribbean men must be interpreted with care, as the possibility of a chance association cannot be ruled out.

Our study is subject to several limitations inherent to the case—control design used. Factors potentially generating bias must be considered, particularly those relating to differential errors in disease assessment. In both populations, cases were identified on the basis of unambiguous histological criteria and our analysis was limited to incident cases during the study period. Controls were selected on the basis of normal findings on digital rectal examination and PSA levels in the normal range for age, taking into account the ethnic background of the population. In some cases (benign prostatic hyperplasia) the absence of malignancy was confirmed histologically. We cannot exclude the possibility that some control individuals had latent disease not detectable by PSA analysis or digital rectal examination. Undetected prostate cancer in control subjects would be expected to bias estimates toward the null, resulting in an underestimation of positive associations. In Guadeloupe, we included incident cases from a recruitment area covering the whole of Guadeloupe, and controls were selected from a representative sample of the male Guadeloupean population during the study period. In Kinshasa, the study was hospital-based for both cases and controls, limiting the possibility of generalizing the results obtained to the total Congolese population. The differential misclassification of genotypes with respect to case status is unlikely, because the individuals responsible for genotyping were blind to the case/control status of subjects. Finally, we did not apply adjustments for multiple comparisons. Such adjustments are recommended when very many comparisons are simultaneously performed in a hypothesis-free approach. In our study, the number of comparisons was small and the genes and polymorphism were selected at the start of the study. In this situation, any adjustment would reduce the informativeness of an association of possible interest [49]. Furthermore, we did not genotype multiple SNPs and we cannot, therefore, determine the proportion of admixture for the subjects included, by principal component analysis, for example. For this reason, and due to the limitations described above, our results should be considered preliminary and confirmation should be sought through further studies on larger samples.

In summary, our results suggest that some polymorphisms of genes involved in estrogen metabolism, particularly *COMT*, are associated with prostate cancer risk in men of African ancestry. Our estimates should be interpreted with caution as sample sizes were small. Confirmation of these observations in other and larger populations of African origin and mechanistic studies are required before the establishment of a causal link in this ethnic group.

## Supporting Information

S1 Table Detailed *CYP19* genotypes and alleles frequencies in cases and controls. (DOCX)

S2 Table Detailed *UGT1A1* genotypes and allele frequencies in cases and controls. (DOCX)

S3 Table Genotype and allele frequency comparison in control subjects. (DOCX)
