# Supplementary material for: Polymorphisms of Estrogen Metabolism-Related Genes and Prostate Cancer Risk in Two Populations of African Ancestry
Source: PLoS One. 2016 Apr 13;11(4):e0153609. doi: 10.1371/journal.pone.0153609 (PMC4830606; doi:10.1371/journal.pone.0153609)
Supplement: S1 Table — (DOCX) [file pone.0153609.s001.docx]

**Table S1**

**Detailed *CYP19* (rs60271534) genotypes and alleles frequencies in cases and controls**

|  | **Afro-Caribbean** | | **Native African** | |
| --- | --- | --- | --- | --- |
|  | **Cases**  **Frequency (*n*)** | **Controls**  **Frequency (*n*)** | **Cases**  **Frequency (*n*)** | **Controls**  **Frequency (*n*)** |
| ***Genotypes*** | | | | |
| 5/6 | 0 (0) | 0.002 (1) | 0 (0) | 0.014 (2) |
| 5/7 | 0.002 (1) | 0 (0) | 0.007 (1) | 0.014 (2) |
| 6/6 | 0.104 (59) | 0.128 (69) | 0.048 (7) | 0.058 (8) |
| 6/7 | 0.286 (162) | 0.253 (136) | 0.299 (44) | 0.225 (31) |
| 6/8 | 0.032 (17) | 0.030 (16) | 0.048 (7) | 0.022 (3) |
| 6/10 | 0.006 (3) | 0.002 (1) | 0 (0) | 0 (0) |
| 6/11 | 0.086 (46) | 0.079 (45) | 0.048 (7) | 0.043 (6) |
| 6/12 | 0.019 (10) | 0.026 (15) | 0.014 (2) | 0.014 (2) |
| 6/13 | 0.002 (1) | 0.002 (1) | 0 (0) | 0 (0) |
| 7/7 | 0.246 (132) | 0.222 (126) | 0.306 (45) | 0.333 (46) |
| 7/8 | 0.045 (24) | 0.039 (22) | 0.048 (7) | 0.072 (10) |
| 7/10 | 0.004 (2) | 0.005 (3) | 0 (0) | 0.007 (1) |
| 7/11 | 0.104 (56) | 0.115 (65) | 0.082 (12) | 0.116 (16) |
| 7/12 | 0.034 (18) | 0.034 (19) | 0.075 (11) | 0.058 (8) |
| 7/13 | 0.002 (1) | 0 (0) | 0 (0) | 0.007 (1) |
| 8/10 | 0 (0) | 0.002 (1) | 0.007 (1) | 0 (0) |
| 8/11 | 0.011 (6) | 0.011 (6) | 0 (0) | 0 (0) |
| 8/12 | 0 (0) | 0.004 (2) | 0 (0) | 0 (0) |
| 9/11 | 0 (0) | 0.002 (1) | 0 (0) | 0 (0) |
| 10/11 | 0 (0) | 0 (0) | 0 (0) | 0.007 (2) |
| 11/11 | 0.021 (12) | 0.024 (13) | 0.014 (2) | 0.007 (2) |
| 11/12 | 0.014 (8) | 0.002 (1) | 0.007 (2) | 0 (0) |
| 12/12 | 0.004 (2) | 0.002 (1) | 0 (0) | 0.007 (2) |
| ***Alleles*** | | | | |
| 5 | 0.001 (1) | 0.001 (1) | 0.003 (1) | 0.011 (3) |
| 6 | 0.328 (352) | 0.316 (358) | 0.252 (74) | 0.217 (60) |
| 7 | 0.467 (501) | 0.462 (524) | 0.561 (165) | 0.583 (161) |
| 8 | 0.044 (47) | 0.042 (47) | 0.051 (15) | 0.047 (13) |
| 9 | 0 (0) | 0.001 (1) | 0 (0) | 0 (0) |
| 10 | 0.005 (5) | 0.004 (5) | 0.003 (1) | 0.011 (3) |
| 11 | 0.126 (135) | 0.131 (149) | 0.082 (24) | 0.087 (24) |
| 12 | 0.029 (31) | 0.042 (48) | 0.048 (14) | 0.036 (10) |
| 13 | 0.002 (2) | 0.001 (1) | 0 (0) | 0.004 (1) |

*n*: total number of individuals and total number of chromosomes for genotype frequency and allele frequency, respectively.
