# Supplementary material for: Polymorphisms of Estrogen Metabolism-Related Genes and Prostate Cancer Risk in Two Populations of African Ancestry
Source: PLoS One. 2016 Apr 13;11(4):e0153609. doi: 10.1371/journal.pone.0153609 (PMC4830606; doi:10.1371/journal.pone.0153609)
Supplement: S2 Table — (DOCX) [file pone.0153609.s002.docx]

**Table S2**

**Detailed *UGT1A1* *(*rs8175347) genotypes and allele frequencies in cases and controls**

|  | **Afro-Caribbean** | | **Native African** | |
| --- | --- | --- | --- | --- |
|  | **Cases**  **Frequency (*n*)** | **Controls**  **Frequency (*n*)** | **Cases**  **Frequency (*n*)** | **Controls**  **Frequency (*n*)** |
| ***Genotypes*** |  |  |  |  |
| 5/5 | 0.011 (6) | 0.011 (6) | 0.007 (1) | 0 (0) |
| 5/6 | 0.049 (27) | 0.058 (33) | 0.068 (10) | 0.080 (11) |
| 5/7 | 0.047 (26) | 0.058 (33) | 0.048 (7) | 0.109 (15) |
| 5/8 | 0.009 (5) | 0.004 (2) | 0.007 (1) | 0.022 (3) |
| 6/6 | 0.266 (146) | 0.229 (131) | 0.231 (34) | 0.196 (27) |
| 6/7 | 0.337 (185) | 0.415 (236) | 0.361 (53) | 0.304 (42) |
| 6/8 | 0.047 (26) | 0.037 (21) | 0.027 (4) | 0.022 (3) |
| 7/7 | 0.197 (108) | 0.159 (91) | 0.197 (29) | 0.210 (29) |
| 7/8 | 0.035 (19) | 0.030 (17) | 0.048 (7) | 0.058 (8) |
| 8/8 | 0.002 (1) | 0 (0) | 0.007 (1) | 0 (0) |
| ***Alleles*** |  |  |  |  |
| 5 | 0.064 (70) | 0.070 (80) | 0.068 (20) | 0.105 (29) |
| 6 | 0.483 (530) | 0.484 (469) | 0.459 (135) | 0.399 (110) |
| 7 | 0.406 (446) | 0.411 (40) | 0.425 (125) | 0.446 (123) |
| 8 | 0.047 (52) | 0.035 (40) | 0.048 (14) | 0.051 (14) |

n: total number of individuals and total number of chromosomes for genotype frequency and allele frequency, respectively.
