# Supplementary material for: Polymorphisms of Estrogen Metabolism-Related Genes and Prostate Cancer Risk in Two Populations of African Ancestry
Source: PLoS One. 2016 Apr 13;11(4):e0153609. doi: 10.1371/journal.pone.0153609 (PMC4830606; doi:10.1371/journal.pone.0153609)
Supplement: S3 Table — (DOCX) [file pone.0153609.s003.docx]

**Table S3**

**Genotype and allele frequency comparison in control subjects**

|  | **Afro-Caribbean**  ***n* (%)** | **Native African**  ***n* (%)** | ***P* value ^a^** |
| --- | --- | --- | --- |
| ***CYP17* (rs743572)** |  |  |  |
| ***Genotypes*** |  |  |  |
| T/T | 208 (39.1) | 50 (36.0) | 0.41 |
| T/C | 265 (49.8) | 68 (48.9) |  |
| C/C | 59 (11.1) | 21 (15.1) |  |
| ***Alleles*** |  |  |  |
| T | 681 (64.5) | 168 (60.4) | 0.21 |
| C | 375 (35.5) | 110 (39.6) |  |
| ***CYP19* (rs60271534)** | |  |  |
| ***Genotypes*** |  |  |  |
| ≤7/≤7 | 329 (63.0) | 89 (64.5) | 0.21 |
| ≤7/>7 | 168 (32.2) | 47 (34.1) |  |
| >7/>7 | 25 (4.8) | 2 (1.4) |  |
| ***Alleles*** |  |  |  |
| ≤7 | 826 (79.1) | 225 (81.5) | 0.38 |
| /≤7 | 218 (20.9) | 51 (18.5) |  |
| ***CYP1B1* (rs1056836)** | |  |  |
| ***Genotypes*** |  |  |  |
| C/C | 42 (7.7) | 3 (2.2) | 0.01 |
| C/G | 205 (37.4) | 43 (30.9) |  |
| G/G | 301 (54.9) | 93 (66.9) |  |
| ***Alleles*** |  |  |  |
| C | 289 (0.26) | 49 (0.18) | 0.002 |
| G | 807 (0.74) | 229 (0.82) |  |
| ***COMT* (rs4680)** |  |  |  |
| ***Genotypes*** |  |  |  |
| G/G | 229 (41.8) | 69 (49.6) | 0.21 |
| G/A | 254 (46.3) | 58 (41.7) |  |
| A/A | 65 (11.9) | 12 (8.6) |  |
| ***Alleles*** |  |  |  |
| G | 712 (0.65) | 196 (0.71) | 0.08 |
| A | 384 (0.35) | 82 (0.29) |  |
| ***UGT1A1 (*rs8175347)** | |  |  |
| ***Genotypes*** |  |  |  |
| <6/<6 | 161 (30.6) | 38 (27.5) | 0.05 |
| <6/>6 | 266 (44.6) | 63 (45.7) |  |
| >6/>6 | 99 (18.8) | 37 (26.8) |  |
| ***Alleles*** |  |  |  |
| <6 | 477 (54.7) | 139 (50.4) | 0.21 |
| <6 | 395 (45.3) | 137 (49.6) |  |

*^a^* P values were calculated in two-tailed Chi2 tests for comparisons of percentages

*n*: total number of individuals and total number of chromosomes for genotype frequency and allele frequency, respectively.
